# Congenital Right Coronary Artery Fistula Causing an Aortic Steal: A Rare Anatomic Abnormality and a Review of the Literature

**DOI:** 10.7759/cureus.11084

**Published:** 2020-10-21

**Authors:** Jason D Vadhan, Gina S Gilderman, Ileana Fuentes

**Affiliations:** 1 Neurosurgery, College of Osteopathic Medicine, Nova Southeastern University, Miami, USA; 2 Osteopathic Medicine, Burrell College of Osteopathic Medicine, University Park, USA; 3 Pediatrics, Borinquen Medical Center, Miami, USA

**Keywords:** congenital, cardiology, coronary artery, coronary artery fistula, cardiac anatomy

## Abstract

Coronary artery fistulas are an uncommon anatomic anomaly with variable presentations. We present an asymptomatic seven-month-old black male with a large coronary artery fistula draining into the right ventricle, causing an aortic backflow on diastole. Despite this prominent alternative drainage pathway, coronary fistulas are commonly an incidental finding and, as in this case, may not require intervention. Through an assessment of previous literature, we recommend providers maintain an elevated index of suspicion for coronary artery fistulas in young persons who present with signs of heart failure, and that the decision to treat should be determined based on the patient's symptoms, age at presentation, and imaging finding severity

## Introduction

Coronary artery abnormalities are exceedingly uncommon, with the incidence of coronary anomalies being approximately 0.23% [1]. Similarly, the incidence of coronary fistulas is believed to be even less. As with many vascular derivations, coronary artery fistulas may be acquired or congenital and can present with additional cardiac abnormalities [2]. The clinical significance of a coronary fistula's is determined by the extent of the hemodynamic sequelae. Situations that cause aberrant coronary blood flow away from other vessels, including the aorta, are known as a coronary or aortic steal. In these cases, localized blood flow reversal can precipitate myocardial or peripheral ischemia independent of structural stenosis or high output heart failure [3]. To date, 18 previous cases of congenital coronary artery fistulas have been reported since 1993.

We present the case of an asymptomatic patient with a right coronary artery fistula draining into the right ventricle, which did not require intervention. We also performed a systematic review of the literature of all reported cases of congenital coronary artery fistulas with an exploration of management techniques.

## Case presentation

A seven-month-old male born full-term via cesarean secondary to failure to progress presented for a well-child visit. Family history was only notable for a maternal grandmother who reportedly had a "hole in her heart” that closed when she was young.

His vital signs were within normal limits. His cardiovascular exam was notable for a harsh holosystolic murmur noted along the left sternal border with radiation to the back. S1/S2 was normal with regular rate and rhythm. There were no S3, S4 rubs, or gallops. Pulses were 2+, and capillary refill took < 3 seconds. Extremities demonstrated no clubbing, cyanosis, or edema, and no signs of volume overload.

A 12-lead electrocardiogram demonstrated sinus rhythm, normal axis, regular intervals, and voltage appropriate for age. The echocardiogram did not reveal any detectable ischemia or wall motion abnormalities. A patent foramen ovale and a proximal right coronary arterial ectasia measuring 22 mm were detected. The ectasia demonstrated an appreciable coronary cameral fistula from the distal right coronary artery to the right ventricle. As a result of the prominent pathway from the right coronary artery feeding into the right ventricle, an abdominal aortic Doppler ultrasound demonstrated a reversal of blood flow during the diastolic phase of the cardiac cycle, most likely due to drainage from the right coronary artery fistula into the right ventricle. Finally, there was a restrictive posterior-inferior muscular ventricular septal defect, with a pressure gradient between 60 to 65 mmHg across the defect. Despite the flow reversal and ventricular septal defect, the right ventricular pressure and volume remained within normal limits, with close monitoring for the fistula recommended.

Upon two months follow-up, the patient remained asymptomatic without any signs of failure to thrive. Vital signs were within normal limits. The cardiovascular exam demonstrated a regular rate and rhythm, normal S1 and S2, 2+ pulses, and a capillary refill of < 3 seconds. A 2/6 short systolic murmur along the left lower sternal border and a harsh continuous murmur along the left sternal border with radiation to the back were notable.

A repeat 12-lead electrocardiogram demonstrated normal sinus rhythm, normal axis, intervals, and voltage appropriate for age. No detectable ischemia was present. No pathologic Q waves or ST-segment changes were noted. A repeat Doppler echocardiogram again demonstrated proximal right coronary arterial ectasia (measuring 23 mm) with a cameral coronary fistula from the distal right coronary artery to the right ventricle. However, there was now no appreciable diastolic flow reversal in the abdominal aortic Doppler. The restrictive posterior-inferior muscular ventricular septal defect remained, demonstrating a gradient of 65 mmHg across the defect, suggesting normal right ventricle pressures and no indication of right heart failure or cor pulmonale. A computed tomography angiogram confirmed a tortuous mid-segment right coronary artery fistula draining into the right ventricle. The left coronary artery demonstrated no abnormalities. No intervention was indicated for this patient since the aortic steal ceased, and that there were no clinically appreciable findings despite a persistent coronary fistula. The patient has continued to do well upon 30 months of follow-up, with no signs of right ventricular volume overload, cor pulmonale, or congestive heart failure.

## Discussion

The PubMed database and all major pediatric and cardiology journals were searched during October of 2020 using the keywords “coronary artery fistula,” “congenital,” “coronary ectasia,” and “congenital cardiac abnormality,” alone or in combination to obtain articles fitting the inclusion and exclusion criteria. The inclusion criteria were congenital coronary artery involving any coronary artery with drainage into either the atria or the ventricle. Acquired coronary fistulas, as well as congenital coronary abnormalities not draining into a cardiac chamber, were excluded.

Associations among quantitative variables were assessed using Pearson’s product-moment correlation coefficient. Associations between categorical variables were evaluated using a Chi-square test or Fisher’s exact test of independence.

To date, only 18 cases of congenital coronary artery fistulas with drainage into the cardiac chamber have been reported since 1993, excluding this case (Table 1). The outcomes of these cases were excellent, with all patients reporting survival following discovery and intervention. The age of discovery spanned from birth to 71 years old, with the majority presenting under 10 years of age.

**Table 1:** Summary of Reported Cases of Congenital Coronary Artery Fistulas RCA: Right Coronary Artery, LCA: Left Coronary Artery, LAD: Left Anterior Descending Artery, LCX: Left Circumflex Artery, RV: Right Ventricle, LV: Left Ventricle, RA: Right Atria, LA: Left Atria, CHF: Congestive Heart Failure, RBBB: Right Bundle Branch Block, VSD: Ventricular Septal Defect, ASD: Atrial Septal Defect, PDA: Patent Ductus Arteriosus, PFO: Patent Foramen Ovale

Author, Year	Patient's Age at Discovery (Months)	Congenital?	Coronary Artery	Fistula Terminal Drain Site	Maximum Diameter of Fistula (mm)	Presenting Symptom	Additional Cardiac Abnormalities	Interventions	Patient's Status on Follow-Up	Length of Follow-Up (Months)
Aggarwal et al. (2018) [4]	0	Yes	RCA, LCA	RV	16	Tachypnea and tachycardia	None	Intravascular Repair: Transcatheter closure via Gianturco coils	Improved	144
Aggarwal et al. (2018) [4]	0	Yes	LAD	LV	16	Incidental	None	Intravascular Repair: Amplatzer Vascular Plug type-II and Micro-Vascular plugs	N/A	7
Waqar et al. (2018) [5]	96	Yes	RCA	RV	N/A	Dyspnea and palpitations	None	Surgical Repair: Pericardial patch on cardiopulmonary bypass	Improved	N/A
Benlafqih et al. (2007) [6]	672	Yes	LCX	RA	15	CHF	None	Surgical Repair: Ligation	Improved	6
Ishii et al. (2010) [7]	72	Yes	LCA	LV	5	Incidental	None	Surgical Repair: Ligation	N/A	3
Tanaka et al. (1997) [3]	852	Yes	RCA	LV	N/A	Dyspnea and Palpitations	None	Surgical Repair: Symbas's operation, cardiopulmonary bypass	Improved	23
Yoldas et al. (2019) [8]	14	Yes	RCA	RV	N/A	Incidental	Absent LCA	None	N/A	N/A
Taha et al. (2018) [9]	816	N/A	N/A	LV	N/A	Incidental	Takotsubo cardiomyopathy	Medical Management	N/A	N/A
Gribaa et al. (2014) [10]	324	Yes	LAD	RV	3	RBBB	VSD, pulmonic stenosis	Intravascular Repair: Transcatheter closure of fistula + pulmonary dilatation	Improved	9
Jaswal et al. (2020) [11]	36	Yes	RCA	RV	N/A	Failure to thrive, tachypnea, hepatomegaly, pedal edema	Bicuspid pulmonary valve	Surgical Repair: Ligation	Improved	N/A
Jiang et al. (2012) [12]	684	N/A	RCA	LV	N/A	Chest pain and dyspnea	None	Intravascular Repair: Transcatheter occlusion	Improved	6
Ismail et al. (2012) [13]	11	Yes	RCA	RV	4.5	Incidental	None	Intravascular Repair: Transcatheter occlusion	N/A	1
Burri et al. (2012) [14]	0.25	Yes	LAD	RV	N/A	Dyspnea and cyanosis	VSD, ASD, PDA	Surgical Repair: Ligation	Improved	1.5
Liu et al. (2011) [15]	624	Yes	LCA	LV	N/A	Palpitations	None	Surgical Repair: Ligation	Improved	N/A
Ephrem (1993) [16]	36	Yes	RCA	RV	N/A	Incidental	None	Surgical Repair: Ligation	N/A	N/A
Guarnera et al. (1994) [17]	0	Yes	RCA	RV	N/A	Incidental	None	None	N/A	3
Yilmazer et al. (2014) [18]	0.5	Yes	LCA	RV	3.3	Incidental	None	None	N/A	10
Jung et al. (2007) [19]	0	Yes	LAD	LV	4.4	Incidental	VSD, PFO	Medical Management	N/A	N/A
Vadhan et al. (2020) [this case]	0	Yes	RCA	RV	22	Incidental	VSD, PFO	None	N/A	30

**Figure 1:**
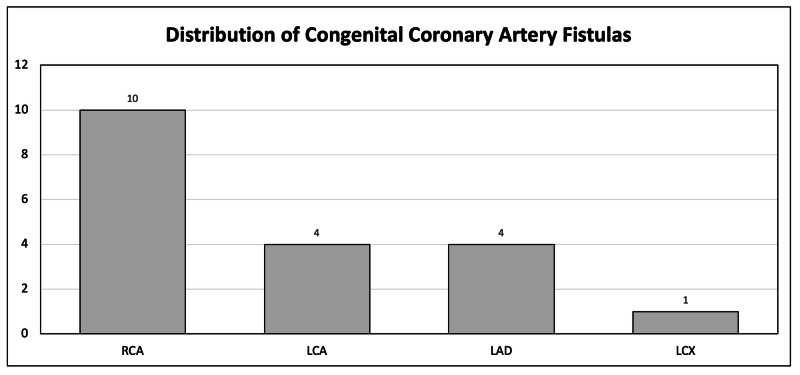
Distribution of Coronary Artery Fistulas RCA: Right Coronary Artery, LCA: Left Coronary Artery, LAD: Left Anterior Descending Artery, LCX: Left Circumflex Artery. The most common location of a congenital coronary artery fistula is the right coronary artery.

The most common coronary artery involvement was the right coronary artery (Figure 1), which was the artery of interest in our case report. Interventions included observation, medical management, surgical intervention, and intravascular catheterization.

Congenital right coronary artery fistula with drainage into the right ventricle is an extraordinarily uncommon finding, as this is only the 19th reported case in the literature to date. Coronary artery fistulas can arise anywhere within the cardiopulmonary circulatory pathway, but most commonly arise in the right coronary artery. As was the case with this patient, they can drain into the right ventricle and create a left to right shunt. The resulting hyperdynamic system can induce cor pulmonale, isolated right heart failure, or even high-output heart failure. Occasionally, an aortic steal phenomenon may also occur. This physiologic pathway is closely tied to the pressure gradient, originating from the coronary vasculature and continuing into the receiving ventricle. If the fistula is large, the intracoronary diastolic pressure progressively diminishes [20]. In the case of this patient, the presence of a prominent accessory network reduces afterload and the total amount of blood in peripheral circulation, which results in a reduction in blood flow, ultimately accentuating the diastolic backflow.

Our assessment highlights several patterns regarding the presentation and treatment of these tumors. First, coronary artery fistulas are most commonly discovered before the age of 10 (68.42%); however, the mean age of discovery is 18.58 years (95% CI: 6.48 to 30.69). Second, most patients presented asymptomatically; however, the most common presenting symptoms relate to signs of heart failure, with the exception of one paradoxical case in which the patient presented with right bundle branch block. Third, with regards to intervention, options include medical management, intravascular correction, or surgical correction. Not all instances require such intervention (as is the case in this patient).

Interestingly, our assessment determined that there was no correlation between fistula diameter and symptomatology, most likely due to the high rate of discovery in the neonatal period, which limits the chance of developing sequelae. There was also no correlation between presenting symptom or symptom severity, and the decision to intervene (p = 0.1273). Lastly, there was no correlation between the age of discovery and the decision to intervene (p = 0.2554). Ultimately, treating this condition should be made based on the symptom severity, the age at discovery, and the extent of the hemodynamic compromise. Given this, we concluded that the patient described was not a surgical candidate, despite it being the largest fistula reported to date.

## Conclusions

Congenital coronary artery fistulas are rare, with a good prognosis, and most commonly present in an asymptomatic child. Occasionally, the resulting prominent alternative hemodynamic pathway can result in a transient aortic backflow during diastole, known as an aortic steal. Although surgical or intravascular interventions are available, it is not always required. Following our evaluation of the data, we recommend that providers maintain an elevated index of suspicion for coronary artery fistulas in young persons who present with signs of heart failure outside the realm of ischemia and that the decision to treat should be determined based on the patient's symptoms, age at presentation, and imaging finding severity
